# The role of leisure traveling in supporting mental health in later life

**DOI:** 10.1192/j.eurpsy.2026.10741

**Published:** 2026-07-31

**Authors:** S. von Humboldt, N. Ilyas, I. Leal

**Affiliations:** 1 William James Center for Research, ISPA – Instituto Universitário, Lisbon, Portugal; 2 Center for Clinical Psychology, University of the Punjab, Lahore, Pakistan

## Abstract

**Introduction:**

Leisure travel has the potential to enhance the emotional and mental health of older adults, who face unique well-being challenges.

**Objectives:**

This study seeks to: a) explore older adults’ emotional experiences related to leisure travel; and b) examine the impact of leisure travel on their mental health.

**Methods:**

A sample of 588 older adults, aged between 65 and 81 years (*M* = 72.7; *SD* = 4.31) and representing three nationalities, participated in this study. Semi-structured interviews were conducted, and content analysis was applied to the collected data.

**Results:**

For the first objective, eight themes emerged: (1) Reduced feelings of loneliness and social isolation (88.3%); (2) Boosted self-confidence (84.9%); (3) Development of meaningful social connections (75.2%); (4) Increased opportunities for physical movement (70.1%); (5) Experiences of personal growth (66.4%); (6) Strengthened sense of autonomy (61.2%); (7) Enhanced ability to manage stress and challenges (59.5%); and (8) Greater overall sense of well-being (57.3%). For the second objective, four themes emerged: (1) Lower depressive symptoms (78.3%), (2) Alleviation of anxiety and stress (77.3%), (3) Improved cognitive adaptability (75.3%), and (4) Better sleep patterns (63.9%). Portuguese older adults primarily emphasized reduced feelings of loneliness and social isolation as well as alleviation of stress and anxiety. English older adults highlighted the development of meaningful social connections and lower depressive symptoms, while Brazilian participants focused on increased opportunities for physical movement and improved cognitive adaptability.

**Conclusions:**

Leisure travel enriches the lives of older adults by boosting mental health, underscoring its important role in supporting comprehensive well-being during later years.

Keywords: Leisure traveling; older adults; mental health; emotional experiences; qualitative study.

**Disclosure of Interest:**

None Declared

